# Binary Mixtures of Imidazolium-Based Protic Ionic Liquids. Extended Temperature Range of the Liquid State Keeping High Ionic Conductivities

**DOI:** 10.3389/fchem.2022.915683

**Published:** 2022-07-01

**Authors:** Iqbaal Abdurrokhman, Anna Martinelli

**Affiliations:** Department of Chemistry and Chemical Engineering, Chalmers University of Technology, Gothenburg, Sweden

**Keywords:** protic ionic liquids, mixtures, imidazolium, vibrational spectroscopy, phase behavior, ionic conductivity

## Abstract

Binary mixtures based on the two protic ionic liquids 1-ethylimidazolium triflate ([C_2_HIm][TfO]) and 1-ethylimidazolium bis(trifluoromethanesulfonyl)imide ([C_2_HIm][TFSI]) have been investigated, with focus on phase behavior, ionic conductivity, and intermolecular interactions as a function of composition (*χ*_*TFSI*_ indicating the mole fraction of the added compound). It is found that on addition of [C_2_HIm][TFSI] to [C_2_HIm][TfO], the melting temperature is first decreased (0 <χ≤ 0.3) and then suppressed (0.3 <χ≤ 0.8) resulting in mixtures with no phase transitions. These mixtures display a wide temperature range of the liquid state and should be interesting for use in devices operating at extreme temperatures. The ionic conductivity does not vary significantly across the composition range analyzed, as evidenced in the comparative Arrhenius plot. The activation energy, E_*a*_, estimated by fitting with the Arrhenius relation in a limited temperature range (between 60 and 140 °C) varies marginally and keeps values between 0.17 and 0.21 eV. These marginal differences can be rationalized by the initially very similar values of the two neat protic ionic liquids. Vibrational spectroscopy, including both Raman and infrared spectroscopies, reveals weakening of the cation–anion interactions for increasing content of [C_2_HIm][TFSI], which is reflected by the blue shift of the average N-H stretching mode and the red shift of the S-O stretching mode in the TfO anion. These trends correlate with the higher disorder in the mixtures observed by DSC and are evidenced by the decrease and suppression of the melting temperature as the amount of [C_2_HIm][TFSI] is increased.

## 1 Introduction

The field of proton exchange membrane (PEM) fuel cells is experiencing a time of revitalized interest and intense research (Jiao et al. (2021); Gittleman et al. (2021)). Progresses with focus on electrodes, catalysts, and electrolytes have been made to improve the performance of the fuel cell device (Wang et al. (2018; 2020)). In the case of PEM fuel cells, the proton-conducting membrane has a crucial role and usually consists of a solid/liquid hybrid material. The state-of-the-art material is a hydrated, perfluorinated polymer that performs best under hydrated conditions and at relatively low temperatures (below 80°C to avoid water evaporation) (Wong et al. (2019); Karimi et al. (2019)). However, materials able to conduct protons under anhydrous conditions and at higher operating temperatures (i.e., above at least 120°C) are desired in order to fit a broader range of applications. In this context, ionic liquids are prominent candidates as new, robust ion- and proton-conducting materials, which can provide good thermal stability as well as high ionic conductivity at anhydrous conditions (Greaves and Drummond (2015)).

Ionic liquids, also known as low-temperature molten salts, are ideally composed of only ions, typically bulky and asymmetric organic cations and anions such that a neat ion packing is prevented and the melting temperature is low. To be classified as ionic liquids, the melting temperature must, by definition, be lower than 100°C. The first ionic liquid was reported in 1914 by Paul Walden (Walden (1914)), who synthesized the archetypical protic ionic liquid ethylammonium nitrate. Since then, and in particular, during the last 20–30 years, the research on ionic liquids has exponentially increased (Lei et al. (2017)) and several sub-classes of ionic liquids are now recognized, such as protic and aprotic ionic liquids, task-specific ionic liquids, and polyionic liquids. In this quickly expanding field, understanding the structure–performance relationship has become pivotal to find pure ionic liquids, hybrid material concepts, or liquid mixtures suitable for specific applications and processes. Ionic liquids have revealed to be multiscale materials, and their behavior in the tiny space of nanopores or nanochannels has become an important field of research, in particular with focus on wall interactions that can fundamentally change the physicochemical properties of nanoconfined ionic liquids (Garaga et al. (2018); Zhang et al. (2017)). Finally, in many chemical reactions and processes, ionic liquids act as efficient solvents and/or catalysts (Dai et al. (2021); Glinska et al. (2021)).

The dual property of high ionic conductivity and low vapor pressure is particularly interesting for using ionic liquids as high-temperature electrolytes in, *e.g.,* fuel cells, but also in supercapacitors and lithium or sodium ion batteries (Stettner and Balducci (2021); Watanabe et al. (2017)). Recent research has also focused on binary mixtures of ionic liquids, either protic or aprotic, and on the possibility of tuning physicochemical properties to target a specific application (Chatel et al. (2014)). A pioneering study of the mixtures of ionic liquids having a common cation but different anions revealed a dependence of properties (for instance the ^1^H NMR chemical shift of the most acidic hydrogen atom on the cation) on the size, symmetry, and coordination ability of the mixed anions (D’Anna et al. (2012)). In another study by Yambou *et al.* (Yambou et al. (2020)), the focus was on mixtures based on aprotic ionic liquids for use in capacitors at low temperatures. The cation was 1-ethyl-3-methylimidazolium, while the three fluorinated anions were FSI^−^, TFSI^−^, and BF_4_^−^. The authors could identify several low-melting mixtures that could stay liquid down to -90°C, implying a potential use extended to unusually low temperatures. Moreover, based on the Walden plot, all mixtures could be classified as “good ionic liquids”. In particular, the mixtures containing the FSI^−^ and the BF_4_^−^ anions displayed superior ionicity, which was explained by the negligible increase in viscosity opposite to the case of mixing with the TFSI^−^ anion instead. Moreover, Kunze et al. (Kunze et al. (2010)) have investigated a number of mixtures based on pyrrolidinium cations and, for instance, FSI^−^ or TFSI^−^ as the anions, finding that the matrix for crystallization is strongly defined by the anions (pure or mixed) hence determining the phase behavior observed by DSC. Very low melting temperatures could be achieved upon mixing, reaching down to −40°C. On the same line but with focus on protic cations, Fumino and co-workers (Fumino et al. (2015)) have investigated the mixing behavior of the protic ionic liquids triethylammonium methylsulfonate [Et_3_NH][CH_3_SO_3_] and triethylammonium triflate [Et_3_NH][CF_3_SO_3_]. The results obtained by far-infrared spectroscopy and molecular dynamics simulations revealed a deviation from ideal mixing, which was explained by a non-ideal H-bond mixing with highly competing anions. Still, in the context of protic ionic liquid mixtures, Miran et al. (Miran et al. (2014)) have reported a study on mixtures based on diethylmethylammonium hydrogensulfate ([dema][HSO_4_] and diethylmethylammonium bis(trifluo-romethanesulfonyl)amide ([dema][NTf_2_]), in which the anions were derived from acids with distinct acidities (i.e. different pKa values). The authors found a lower N-H stretching frequency in [dema][HSO_4_] than in [dema][NTf_2_], revealing that the [HSO_4_]^−^ anion can form stronger hydrogen bonds with the [dema]^+^ cation *via* the NH group. Because of the established hydrogen bonds in the mixtures, the authors propose a proton transport mechanism not only by the vehicular mechanism but also including proton hopping (that is, by the Grotthuss mechanism).

Based on these behaviors, protic ionic liquids and mixtures thereof are closely related to deep eutectic solvents (DES) (Smith et al. (2014)), both classes of materials being of interest for the possibility of tuning properties by an adequate choice of the mixed compounds. One important aspect of research has been the suppression of the melting temperature, and another is the enhancement of transport properties for energy applications (Abbott et al. (2021)). Also, both DESs and protic ionic liquids display extended networks of hydrogen bonds (Low et al. (2019)), which are responsible for many of the observed physicochemical properties. In a way, protic ionic liquids can be seen as the link between more conventional aprotic ionic liquids and DESs (Abbott et al. (2021)). Moreover, DESs are easier to prepare and may be cheaper than protic ionic liquids. Nevertheless, the work presented here focuses exclusively on mixing protic ionic liquids, with the studied binary mixtures having in common the protic cation 1-ethylimidazolium. This cation was selected based on the knowledge that imidazolium-based cations result in higher conductivity and lower viscosity than other cations like for instance those derived from pyrrolidinium or piperidinium. Hence, solutions have been prepared by mixing the two protic ionic liquids 1-ethylimidazolium triflate [C_2_HIm][TfO] and 1-ethylimidazolium bis(trifluoromethylsulfonyl)imide [C_2_HIm][TFSI]; the two anions TfO^−^ and TFSI^−^ deriving from acids of slightly different acidities (pK_*a*_ (TfO) = −11.4 and pK_*a*_ (TFSI) = −12.3 in 1,2-dichloroethane) but also of different size, shape, and symmetry. The TFSI anion is frequently chosen since it typically results in a low-viscosity ionic liquid in combination with many cations; TfO has a related chemical structure but results in a more hydrophilic ionic liquid. Primarily, we have investigated the effect of mixing on phase behavior, ionic conductivity, and strength of intermolecular interactions.

## 2 Materials and Experimental Methods

### 2.1 Materials

**FIGURE 1:**
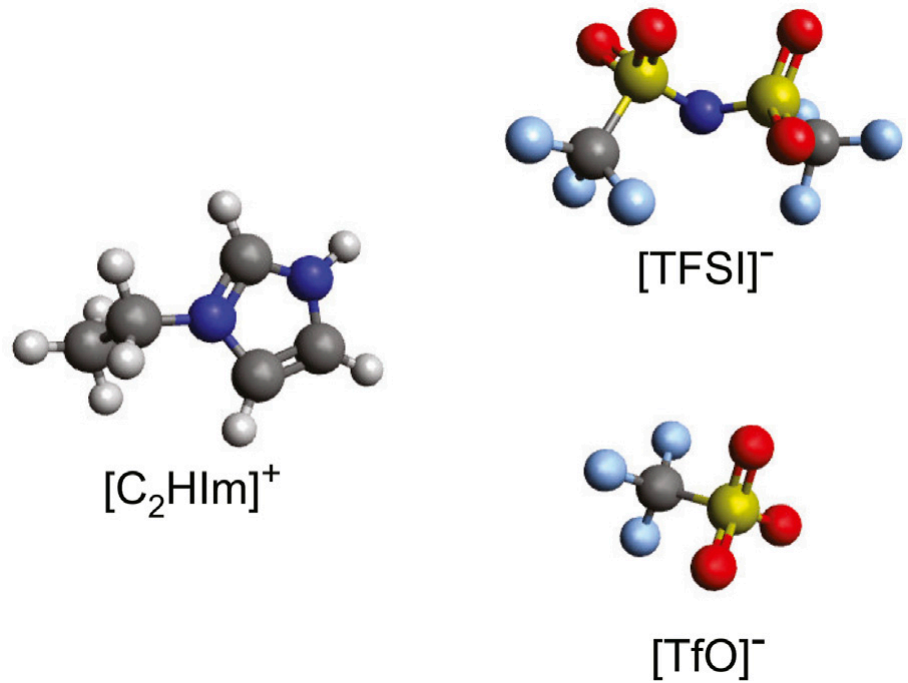
Molecular structure of the 1-ethylimidazolium cation (C_2_HIm^+^), and of the triflate (TfO^−^) and bis (trifluoromethylsulfonyl)imide (TFSI^−^) anions, which are the constituting ions in the two protic ionic liquids investigated. The color code of the atoms is as follows: red, oxygen; gray, carbon; light blue, fluorine; dark blue, nitrogen; and yellow, sulfur.

The protic ionic liquids 1-ethylimidazolium triflate [C_2_HIm][TfO] and 1-ethylimidazolium bis(trifluorome-thylsulfonyl)imide [C_2_HIm][TFSI] were purchased from Io.Li.Tec GmbH, stored inside a nitrogen-filled glovebox and then used without further purification. The molecular structure of the ions constituting these protic ionic liquids is presented in Figure 1. The two protic ionic liquids were mixed at different ratios, resulting in mixtures with a varying mole fraction, *χ*, of [C_2_HIm][TFSI] with *χ* ranging from 0 to 1. The sample preparation was done inside the glovebox in order to prevent absorption of moisture.

### 2.2 Experimental Methods

#### 2.2.1 Differential Scanning Calorimetry

The phase behavior, with respect to melting temperature, crystallization temperature, and glass transition temperature, was determined using a differential scanning calorimetry (DSC) instrument from Mettler Toledo equipped with STARe software. The samples were prepared to have a mass of ca 5–6 mg and were placed in 40 *μ*l sealed aluminum crucibles. The scanning method included an equilibration time of 1 minute at 60°C, followed by cooling the sample down to −100°C at a cooling rate of 20°C/min and a subsequent heating back to 60°C at a rate of 5°C/min. This procedure was repeated twice to analyze the material’s response to subsequent cycles. The sample preparation was done inside a nitrogen-filled glovebox. The melting temperature (T_m_) of all samples was extracted from the minimum of the endothermic peak detected in the DSC curve during the second heating scan.

#### 2.2.2 Vibrational Spectroscopy

Infrared spectroscopy: Infrared spectra were collected using an infrared spectrometer from Perkin-Elmer, in the ATR (Attenuated Total Reflection) mode using a single-reflection diamond crystal from Gladi ATR. The spectra were collected to cover the spectral range 400–4,000 cm^−1^, by 16 cumulative scans and with a resolution of 2 cm^−1^. Nitrogen gas was flowed over the samples during the entire course of the measurements to avoid uptake of moisture by the ionic liquid.

Raman spectroscopy: Raman spectra were recorded at room temperature using a custom-made cell of stainless steel with the sample contained in an internal glass tube. The sample was loaded into this cell inside the glovebox. The Raman spectra were recorded using an InVia Reflex spectrometer from Renishaw, covering the spectral range 100–4,000 cm^−1^. The 785 nm laser was used as the excitation source, setting the power to approximately 10 mW. The spectra were obtained by averaging over 5 scans with an exposure time of 10 s each. The resolution, using a grating with 1,200 grooves per mm, was 2 cm^−1^.

#### 2.2.3 Dielectric Spectroscopy

The ionic conductivity was measured using a broadband dieletric spectroscopy (BDS) instrument from Novocontrol equipped with a cryo-cooling system. The temperature was varied from 20 to 140°C, with a temperature stabilization time of 600 s. The frequency window was chosen to cover the range from 10^–1^–10^7^ Hz. The measurements were done using a low alternating voltage of 10 mV. The samples were placed in a sandwich type of electrochemical cell between two stainless steel electrodes, while the dimensions of the analyzed liquid were kept constant using a Teflon spacer with a diameter of 5 mm and a thickness of 3.1 mm.

## 3 Results and Discussion

### 3.1 Phase Behavior

**FIGURE 2:**
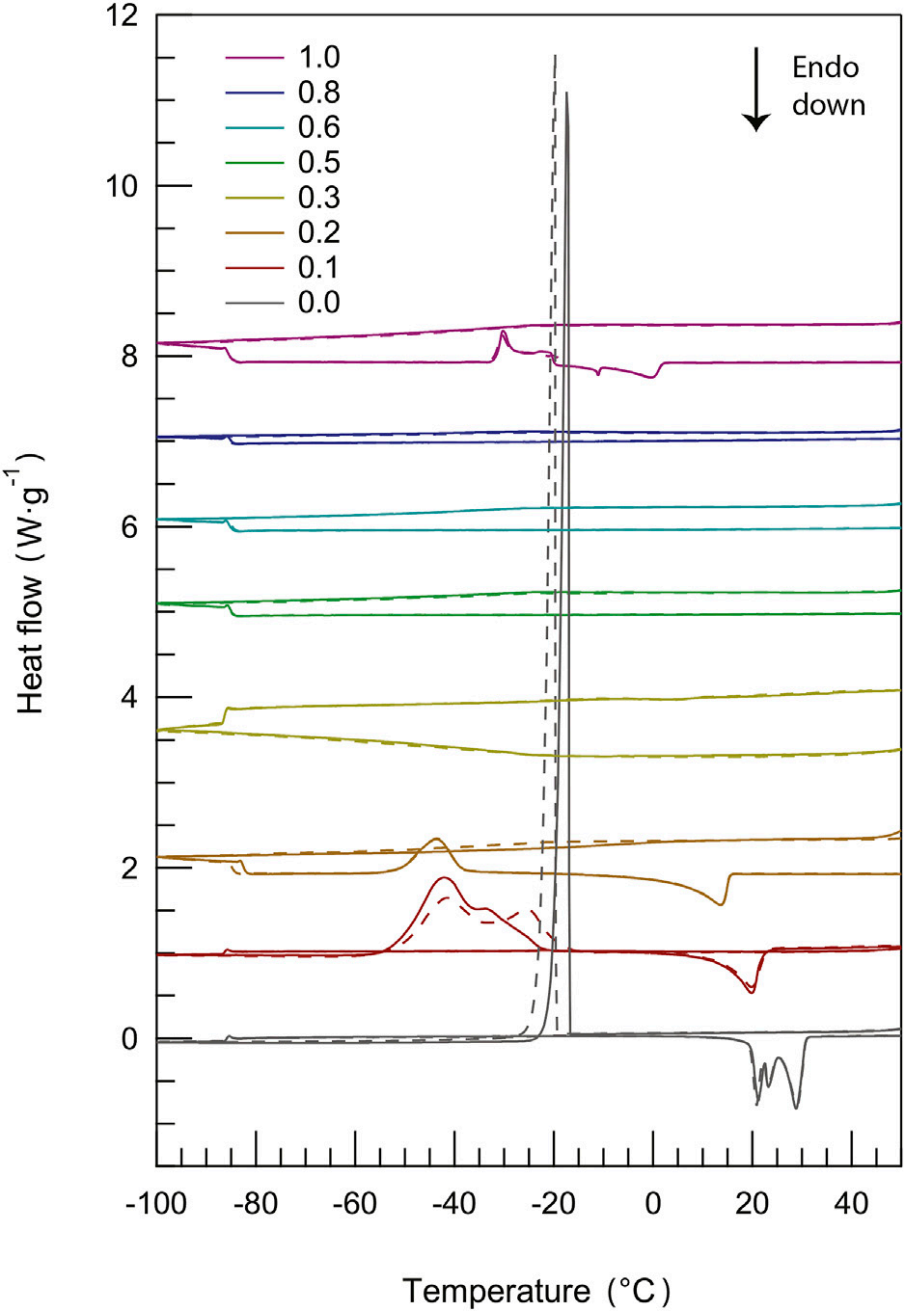
Differential scanning calorimetry (DSC) curves recorded during cooling and heating scans (two cycles) for all protic ionic liquid mixtures, with the content of [C_2_HIm][TFSI] increasing from bottom (*χ* = 0.0) to top (*χ* = 1.0).

The phase behavior of materials can be appropriately studied using methods for thermal analysis, such as differential scanning calorimetry also known as DSC (Gabbott (2008)). By DSC, first-order phase transitions like melting and crystallization as well as glass transitions can be detected and assigned to a specific temperature. In principle, during a DSC measurement, the response of the investigated material as heat is exchanged is measured as a function of temperature (by comparing to the case of an empty cell exposed to the same heating/cooling conditions). This analysis enables distinguishing between exothermic events (e.g., crystallization) and endothermic events (e.g., melting), while a glass transition will appear as a step change in the baseline (i.e. a change in heat capacity). The DSC thermograms recorded for pure protic ionic liquids as well as their binary mixtures during cooling and heating scans are presented in Figure 2 (more detailed information on these DSC scans is available in Supplementary Figure S1). This figure shows that, except for minor shifts, the DSC traces are well reproduced over subsequent cycles. Figure 2 also shows that mixing [C_2_HIm][TFSI] with [C_2_HIm][TfO] has a profound influence on the observed phase behavior.

The pure protic ionic liquid [C_2_HIm][TfO] (*χ* = 0.0) has distinct crystallization (upon cooling) and melting (upon heating) transitions, the latter showing multiple and proximate endothermic peaks. This feature had previously been observed by Yaghini et al. (Yaghini et al. (2015)) for the same ionic liquid and can be attributed to the presence of polymorphic phases (i.e. of energetically close crystal structures). The fact that multiple peaks are not resolved during crystallization is due to the faster rate used upon cooling. In the phase behavior of an ionic liquid of similar structure, that is imidazolium methanesulfonate (Luo et al. (2013)), the multiple endothermic peaks close to melting have been attributed to a plastic crystal phase with some orientational degrees of freedom also involving ${CH}_{3}{SO}_{3}^{-}$ , an anion that has the same symmetry as ${CF}_{3}{SO}_{3}^{-}$ at focus in our study. Upon addition of the second component [C_2_HIm][TFSI] (*χ* = 0.1), both the crystallization peak upon cooling and the melting features upon heating broaden significantly and shift to lower temperatures, as can be expected by an ‘impure’ system. Broadening of the transition peaks and a less sharp melting feature may indicate a wider distribution of crystal sizes and/or an inhomogeneous system. Also, the origin of the double peak observed during crystallization upon cooling is unclear, but may possibly be due to the presence of individual liquid droplets that experience different degrees of supercooling. Such a situation should be verified by eye inspection or complementary techniques, but this was not a priority in our work. In summary, and referring to the report presented by Gomez et al. (Gomez et al. (2018)), pure [C_2_HIm][TfO] and the mixture with *χ* = 0.1 display type-two of the phase behavior.

Upon further addition of [C_2_HIm][TFSI] (*χ* = 0.2), cold crystallization is observed during heating, indicating the unease of the liquid system to form a crystalline structure during cooling. Furthermore, this peak is of lower intensity while the subsequent melting feature is further shifted towards lower temperatures. Still, with reference to the report presented by Gomez et al. (Gomez et al. (2018)), the mixture with *χ* = 0.2 displays type-three of the phase behavior. The mixture with *χ* = 0.3 has a DSC trace equivalent to that of the mixture with *χ* = 0.2, but the peaks associated to the phase transitions (cold crystallization and melting) are of extremely low intensity (see Supplementary Figure S2), and are therefore not visible in the scale used in Figure 2. Interestingly, in the intermediate compositional range where 0.3 < *χ* ≤ 0.8, no first-order phase transitions are observed. Given this scenario, a glass transition may be expected instead, congruent with type-one of the phase behavior (Gomez et al. (2018)). However, this was not the case in our study, implying that the liquid mixtures remain supercooled in the entire temperature range investigated. Finally, for the pure protic ionic liquid [C_2_HIm][TFSI] (*χ* = 1.0) a phase behavior with a cold crystallization directly followed by a broad melting is observed (phase behavior of type-three), as already reported before (Yaghini et al. (2015)). In the case of ionic liquids based on TFSI, the co-existence of two conformations, i.e. the *cisoid* and the *transoid* (Martinelli et al. (2011)), must be considered, which results in different cation–anion structural configurations that may contribute to the width of the melting transition. In addition, benchmarking to DSC results previously reported (Yaghini et al. (2016)), the shape of the cold crystallization and melting peaks suggests the presence of tiny amounts of the (unprotonated) base in the nominally pure protic ionic liquid.

**FIGURE 3:**
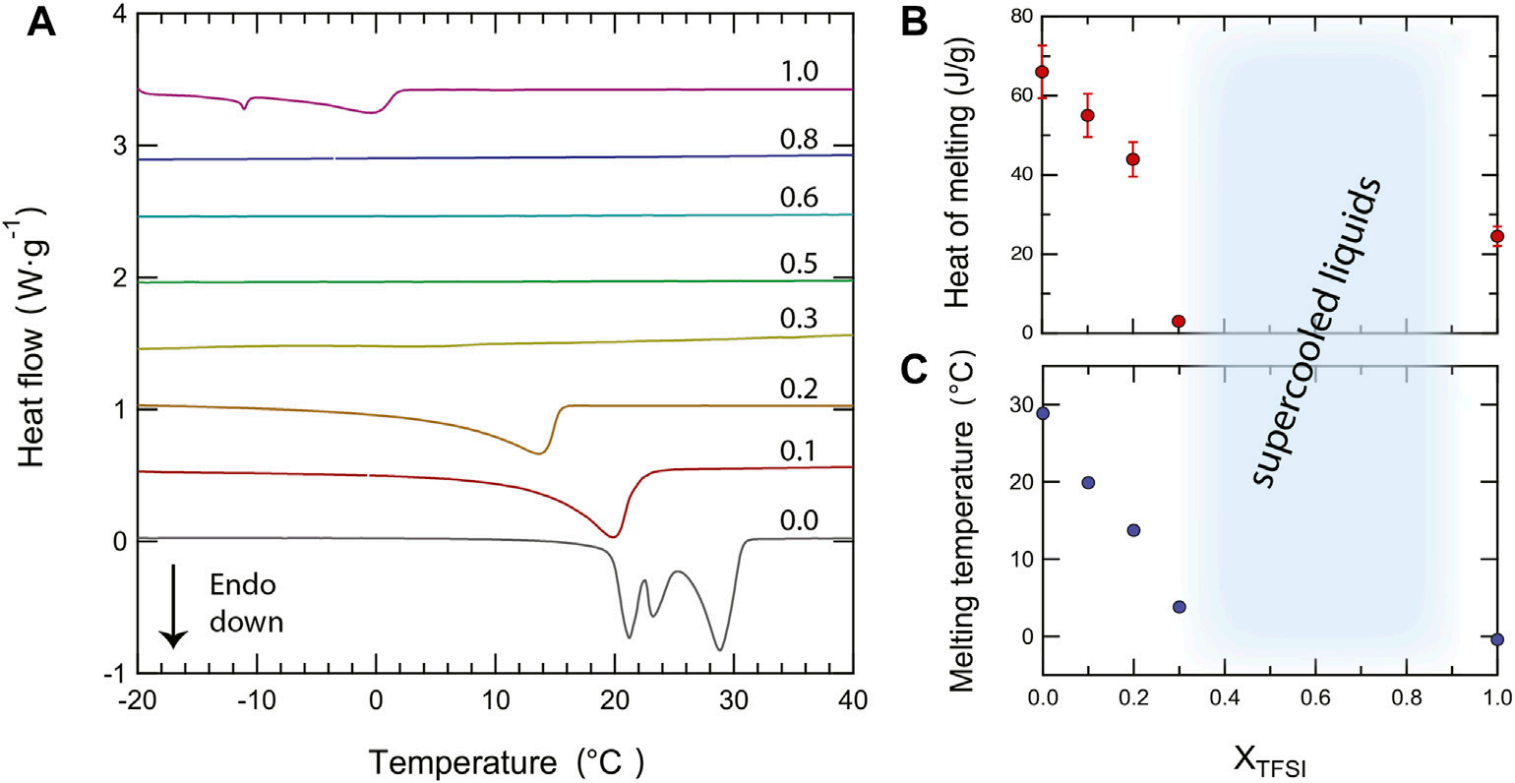
Close-up of the differential scanning calorimetry (DSC) curves in the temperature range of the melting transition (−20—40°C) recorded during the second heating scan for all protic ionic liquid mixtures **(A)**. From bottom to top, traces are shown for an increasing amount of the second component [C_2_HIm][TFSI], given in mole fraction, *χ*, and ranging from 0 to 1. The compositional dependence of the heat of fusion **(B)** and of the melting temperature **(C)** is also shown.

As a complement to these qualitative observations, precise melting temperatures (better visualized in the close-up plot of Figure 3A) and the heat of melting have been estimated from an accurate peak fitting analysis (see Supplementary Figure S3 for an example of the applied procedure). Both the melting temperature and the heat of melting decrease monotonically with the addition of [C_2_HIm][TFSI], at least for *χ* ≤ 0.3, a trend that reflects intermolecular interactions of decreasing strengths, Figure 3B and Figure 3C. This change may be rationalized by considering the intrinsic properties of the anions, TFSI^−^ being a bulkier anion with a larger charge delocalization than TfO^−^, hence involved in weaker cation–anion interactions (which is congruent with the melting temperature of pure [C_2_HIm][TFSI] being lower than that of pure [C_2_HIm][TfO]). Moreover, in the mixtures, local disorder is increased since multiple types of cation–anion interactions can be established (which involve TFSI_*cis*_, TFSI_*trans*_, and TfO); in particular, TFSI provides multiple sites for the formation of hydrogen bonds through the four oxygen atoms and the central nitrogen distributed over the larger molecule, while TfO has the–SO_3_ group as the specific hydrogen bond acceptor site. As a side note, the heat of melting for the neat ionic liquid [C_2_HIm][TfO] is here estimated to be 66 J/g which, considering the molecular weight of this ionic liquid being equal to 246.21 g/mol, translates into ∼16 kJ/mol, a value that is in accordance with the heat of melting of other related ionic liquids such as, for instance, [C_2_C_1_Im][TfO] (∼17 kJ/mol (Valderrama and Cardona (2021)). A gradual decrease of the heat of melting upon addition of a second component to an ionic liquid has previously been observed in a variety of systems, for instance when adding the Li-TFSI salt to [PYR_23_][TFSI] (Martinelli et al. (2009)), water to [C_2_HIm][TfO] (Yaghini et al. (2015)), or imidazole to [C_2_HIm][TFSI] (Yaghini et al. (2016)). On the same line, Elhi *et al.* (Elhi et al. (2020)) have investigated mixtures of ionic liquids based on the choline cation and different carboxylate anions, reporting several eutectic mixtures. In these mixtures, the melting temperature was considerably lower than that of the pure components (the depression of T_*m*_ being as large as 45°C in some cases) and the eutectic behavior was observed at mole fractions of the second component between 0.4 and 0.6. Through this study, the authors observed that a greater depression of the melting point could be achieved by mixing anions of different sizes, e.g. acetate with 2-methylbutyrate. The phase behavior observed in our study for 0.3 < *χ* ≤ 0.8, with a wide temperature range of the liquidus state and a featureless DSC trace, is similar to that observed in mixtures of [C_2_HIm][TFSI] and imidazole for mole fractions of imidazole between 0.17 and 0.8, as reported by Yaghini *et al.* (Yaghini et al. (2016)). Notably, the suppression of crystallization/melting during DSC scans has also been observed upon addition of [C_2_HIm][TFSI] to either [C_2_HIm][BF_4_] or [C_2_HIm][FSI] (Yambou et al. (2020)).

**FIGURE 4:**
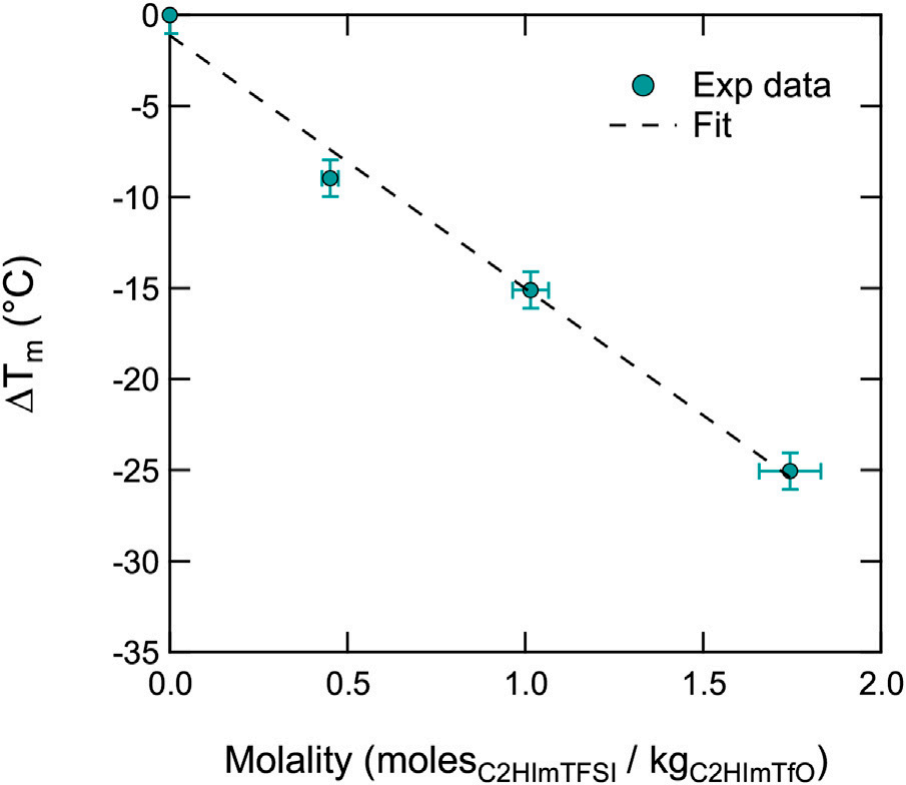
Depression of the melting temperature Tm (of [C2HIm][TfO]) as a function of composition (given in molality).

As a further approach to the experimental data, we have tried to model the depression of the melting point using the well known Van’t Hoff relation ΔT_*m*_ = K⋅i⋅m (Berton et al. (2019)), where K is the cryoscopic constant of the solvent (in this case C_2_HImTfO), i is the dissociation constant of the solute (in this case C_2_HImTFSI), and m is the molality (here moles of C_2_HImTFSI in kilograms of C_2_HImTfO).^1^ Figure 4 shows that this model fits very well the experimental data, at least in the molality range where T_*m*_ has been detected. Also, assuming that i takes a value of 2 (Berton et al. (2019)) by dissociation into one [C_2_HIm] cation and one [TFSI] anion, the linear fit of the experimental data returns a cryoscopic constant for C_2_HImTfO equal to 6.96°C mol^−1^ kg (obtained from the linear slope, that is K⋅i, equal to 13.918 ± 1.06). For comparison, the cryoscopic constant was found to be equal to 6.25°C mol^−1^ kg for trifuoroacetic acid (Harris and Milne (1976)). To the best of our knowledge, the cryoscopic constant of ionic liquids has not been reported nor investigated before, wherefore this result may turn to be an interesting input for further thermal analyses of ionic liquid-based mixtures.

**FIGURE 5:**
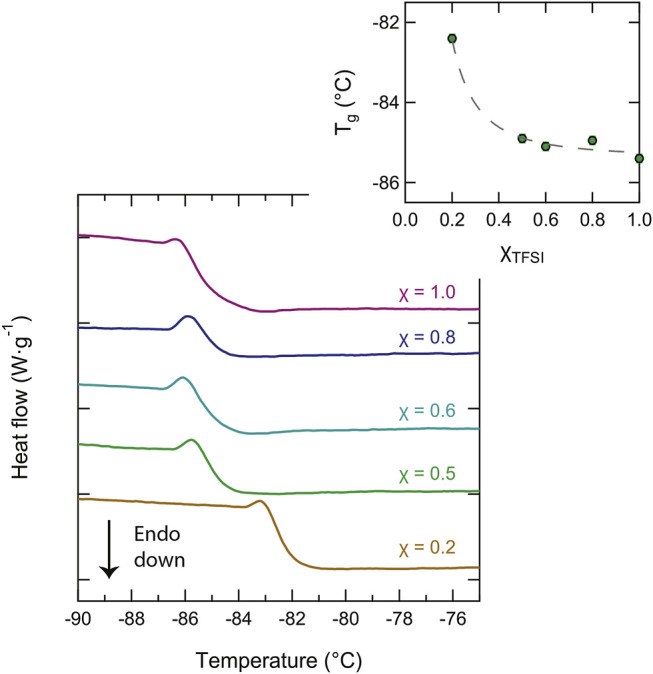
Close-up of the DSC traces showing the low-temperature range where the glass transition temperature, T_*g*_, appears. The dependence of T_*g*_ on composition is shown in the inset plot.

By closer inspection of the DSC traces in the low temperature range, we also found interesting trends for the glass transition temperature, T_*g*_. For certain samples, the glass transition temperature was observed as a clear step change of the baseline during the heating scans, as shown in Figure 5, whereas during cooling, this was not as clear indicating that under the conditions used for fast cooling (20 °C/min), these liquids were supercooled into a non-equilibrium state. The values of T_*g*_ were determined from the mid-point of the step change and were found to be in the range between −86 and −82°C. As the inset plot of Figure 5 shows, these values are slightly dependent on composition, decreasing with an increasing content of the protic ionic liquid [C_2_HIm][TFSI]. This effect is similar to the one observed by Thoms et al. (Thoms et al. (2017)) upon mixing the ionic liquid [BMIM][TFSI] with [BMIM][Cl]. The non-linear change in T_*g*_ with composition suggests the more important role of the bulky TFSI anion in determining T_*g*_. In general, an added component that results in a decrease of T_*g*_ is called a plasticizer.

To conclude, the effect of adding [C_2_HIm][TFSI] to [C_2_HIm][TfO] is, at low molar fractions, to decrease the melting temperature in those mixtures that show a solid–liquid phase transition and, at intermediate molar fractions, to generate mixtures with no phase transitions at all, hence offering a wider temperature range of the liquid state. In addition, from the viewpoint of the glass transition T_*g*_, added [C_2_HIm][TFSI] has a small but measureable plasticizing effect. The most relevant properties achieved from DSC are also given in Table 1. In order to better understand the aspect of intermolecular interactions underpinning these effects, we have used vibrational spectroscopy, as described in more details in the following section.

**TABLE 1:** Temperature of glass transition (T_*g*_), temperature of crystallization (T_*c*_), temperature of melting (T_*m*_), enthalpy of melting (H_*m*_), ionic conductivity (*σ*) at 60 °C, and activation energy (E_*a*_) of the pure liquids and their binary mixtures as a function of composition (*χ*_*TFSI*_).

*χ*_TFSI_	T_*g*_ [°C]	T_*c*_ [°C]	T_*m*_ [°C]	ΔH_*m*_ [J/g]	*σ* [mS/cm]	E_*a*_ [eV]
0.0	—	−16	28.8	66	11.2	0.19
0.1	—	−41; −25	19.9	55	5.4	0.21
0.2	−82.4	−43	13.7	44	10.5	0.20
0.3	—	−8	3.8	3	10.7	0.18
0.5	−84.9	—	—	—	11.2	0.18
0.6	−85.1	—	—	—	11.3	0.18
0.8	−84.9	—	—	—	11.7	0.17
1.0	−85.4	−30	−0.4	24	9.9	0.19

### 3.2 Cation–Anion Interactions and Hydrogen Bonds

**FIGURE 6:**
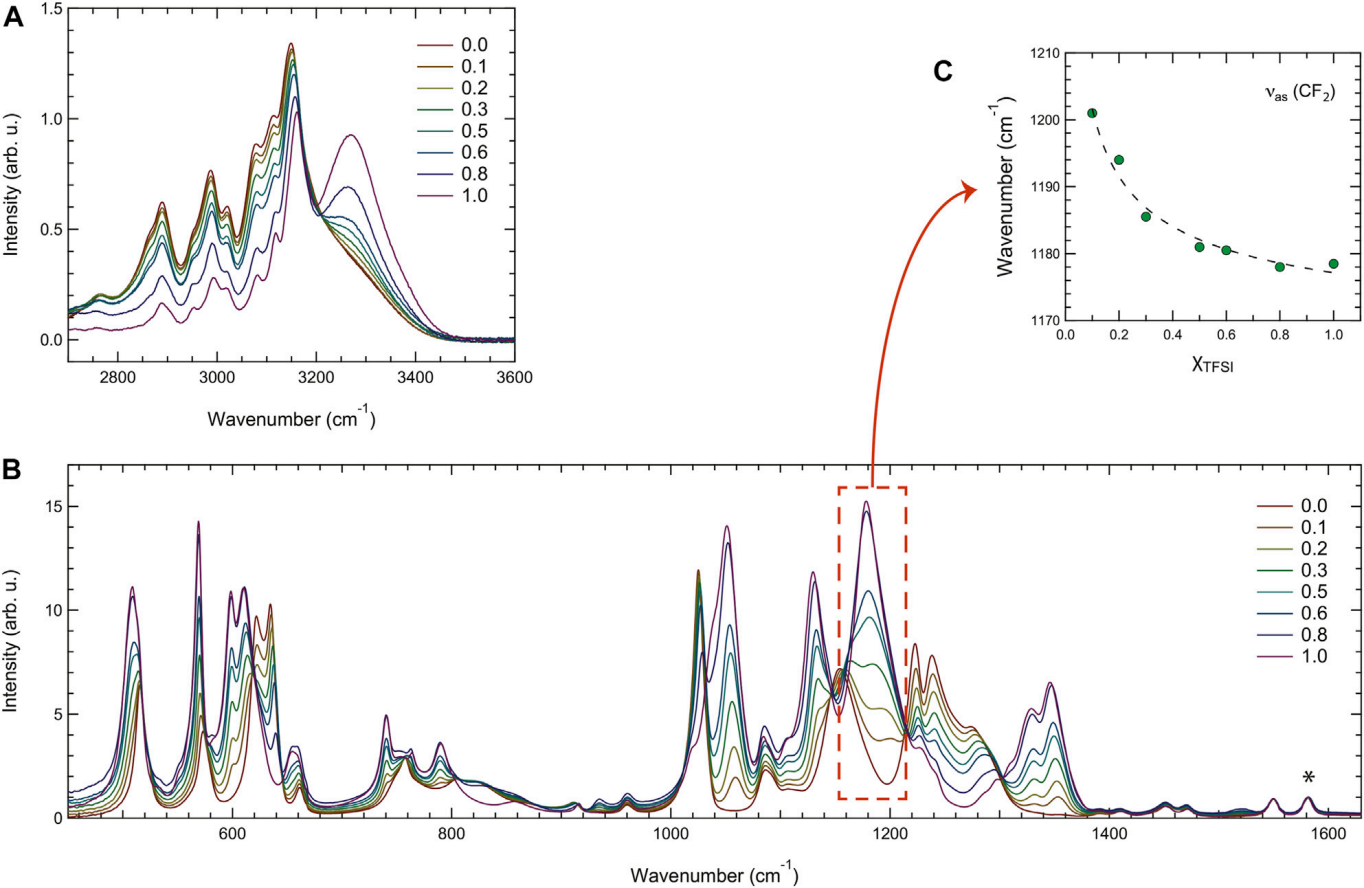
Infrared spectra recorded for all the binary mixtures in the high- **(A)**, and mid- and low-frequency ranges **(B)**. The asterisk in the plot **(B)** indicates the vibrational mode used for normalization. The frequency of the asymmetric stretching mode (*ν*_*as*_) in the CF_3_ group of TFSI as a function of composition is shown in **(C)**.

By vibrational spectroscopy, quantitative and qualitative studies of materials can be performed. The composition and type of intermolecular interactions can be studied, encompassing the strength of hydrogen bonds, presence of ion pairs or ion aggregates, and conformational states. For the specific case of ionic liquids, an extensive review of the use of vibrational spectroscopy has been recently provided by Paschoal et al. (Paschoal et al. (2017)). Although infrared and Raman spectroscopies are usually used in tandem because they probe the same vibrational modes (though filtered by different selection rules), they are based on fundamentally different phenomena; Raman spectra arise from scattered light subsequent to laser–matter interaction while infrared spectra result from absorption (alternatively transmission or reflection) by the matter of light in the infrared spectrum. In this work, vibrational spectroscopy, including Raman and infrared spectroscopies, has been employed to investigate the nature of interactions established between cations and anions within the different liquid mixtures. Figure 6 shows all the infrared spectra in the high frequency region (A) and in the mid-to-low frequency region (B). The spectra are shown after normalization to the mode at 1,580 cm^−1^ (indicated by an asterisk), which is assigned to the in-plane ring deformations of the imidazolium ring and has shown to be independent of the type of anion associated to the cation (Morais et al. (2022)).

Figure 6A includes the C-H stretching modes of the ethyl chain (2,700–3,200 cm^−1^) and the N-H stretching mode of the imidazolium ring (3,200–3,500 cm^−1^). The latter is distinct and peaked at 3,267 cm^−1^ for [C_2_HIm][TFSI], in agreement with a previous spectroscopic investigation (Yaghini et al. (2015)), while for decreasing mole fractions of this ionic liquid, hence approaching the case of pure [C_2_HIm][TfO], the N-H stretching mode red shifts and broadens considerably. These two simultaneous effects may, at least partly, explain why the whole region of the C-H stretching modes appears enhanced in intensity (as if laying over a higher background). The vibrational modes attributed to C-H stretching do not change significantly with composition, as expected, considering that these groups are not in direct interaction with the anions. The spectral range 1,000–1,700 cm^−1^ shown in Figure 6B contains many vibrations assigned to the anions and shows intensity changes that can simply be explained by the varying population of TfO^−^ and TFSI^−^ species. Most of these vibrations shift in frequency with composition, one example being the asymmetric stretching mode of CF_2_ in the CF_3_ group of TFSI^−^ shown in the plot of Figure 6C. The spectral range 400–700 cm^−1^, also shown in Figure 6B, contains mainly vibrations assigned to the bending modes of the CF_3_ and SO_2_/SO_3_ groups of the TFSI^−^ and TfO^−^ anions. Even here, intensities change systematically with the composition.

To get a deeper insight of this spectral evolution, an accurate peak-fit analysis has been applied to the recorded infrared spectra in the high frequency range, an example being illustrated in Supplementary Figure S4. The fitting model was chosen from fitting the spectrum of pure [C_2_HIm][TfO], using the least number of fitting components and keeping the position of all fitting peaks fixed except for the peak describing the N-H stretching, which was free to take any position and width values. Moreover, in the fitted spectral range 2,750–3,600 cm^−1^ a linear background was assumed. This peak-fit analysis reveals an average blue shift of the N-H stretching frequency with an increasing amount of [C_2_HIm][TFSI], confirming the hypothesis that [C_2_HIm]⋅⋅⋅[TFSI] interactions *via* NH⋅⋅⋅anion hydrogen bonding are weaker than those between [C_2_HIm] and [TfO]. This hypothesis was further confirmed by theoretical calculations that reveal a NH^+^⋅⋅⋅O^−^ distance longer for the case of TFSI^−^ (1.95 Å) than TfO^−^ (1.76 Å). However, the NH^+^⋅⋅⋅O^−^ hydrogen bond has a similar angle in the cases of the two anions, as illustrated in the images of Supplementary Figure S5. As shown in Supplementary Figure S6, the frequency change of the N-H stretching mode with the composition is very close to linear and, given the used model with only one component for the N-H stretching mode, this frequency represents an average of all types of cations present in each mixture.

**FIGURE 7:**
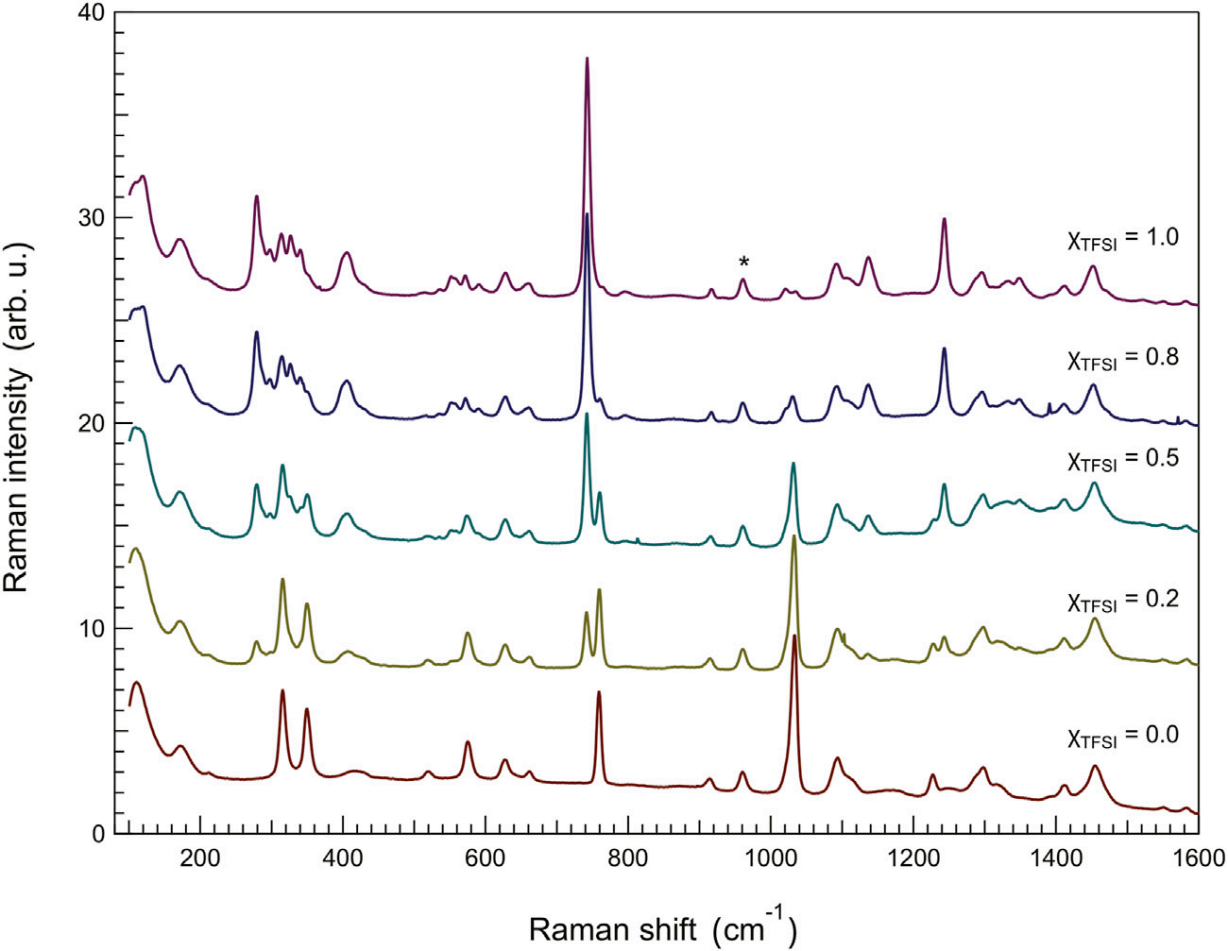
Raman spectra recorded for the binary mixtures, showing the frequency region 80–1,600 cm^−1^. The asterisk shows the vibrational mode used for normalization, that is the mode at 961 cm^−1^ assigned to the C_6_–C_7_ stretching mode in the ethyl chain of the imidazolium cation.

Since the modes most sensitive to intermolecular interactions, from the perspective of the anion, appear stronger in Raman spectroscopy, we have also analyzed the Raman spectra collected for the ionic liquid mixtures as shown in Figure 7. In particular, compared to infrared spectroscopy, Raman spectroscopy provides a higher spectral resolution which allows a more precise distinction of proximate vibrations and a more accurate analysis of frequency shifts. For this analysis, we have focused on two characteristic Raman signatures of the TfO^−^ anion, i.e. the symmetric stretching of -SO_3_ at 1,033 cm^−1^ (*ν*_*s*_SO_3_) and the symmetric stretching (with some contribution from bending as well (Morais et al. (2022)) of -CF_3_ at 759 cm^−1^ (*ν*_*s*_CF_3_). These vibrations have previously been at focus in several computational and experimental studies of material systems containing the Li-triflate salt (Gejji et al. (1993); Hu et al. (2005); Huang et al. (1994); Caruso et al. (2002)). These vibrations are strong in the Raman spectrum and interfere only marginally with modes arising from the TFSI^−^ anion, which simplifies the use and interpretation of peak-fit procedures. Also, the *ν*_*s*_SO_3_ mode is non-degenerate, which allows distinguishing between anionic species in different energetic environments, in particular if splitting was observed (Huang et al. (1994)).

**FIGURE 8:**
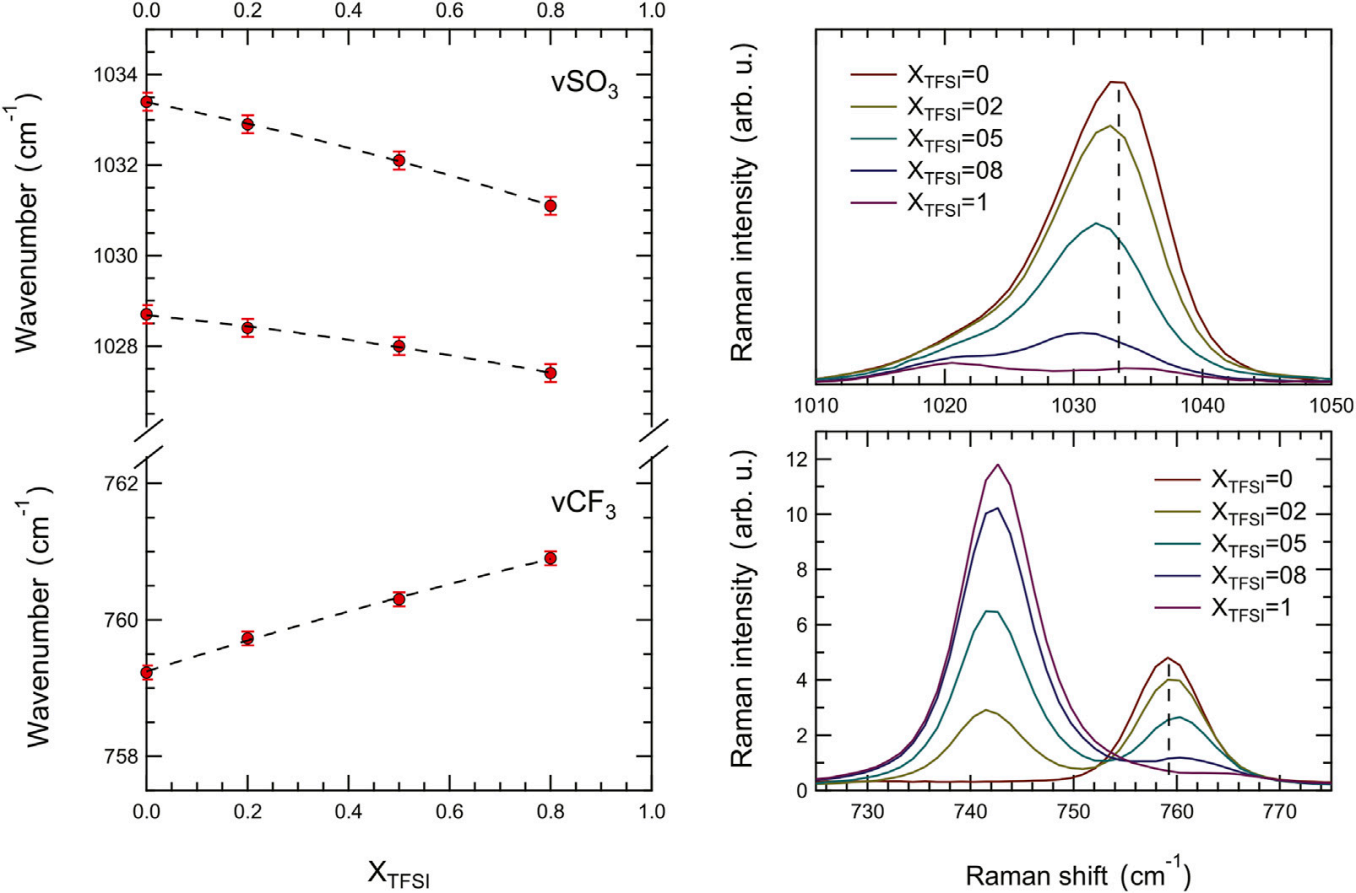
Composition dependence of the Raman frequency shift of the symmetric S-O and C-F stretching modes in the TfO anion.

The results of our peak-fit procedure (see an example in Supplementary Figure S7) reveal a red shift of the main *ν*_*s*_(SO_3_) mode and a blue shift of the *ν*_*s*_(CF_3_) mode for increasing amounts of [C_2_HIm][TFSI], Figure 8. A number of previous studies have focused on these specific modes to understand the type of coordination of the triflate anion (TfO^−^), investigating the case of LiTfO dissolved in solvents or polymers (like polyethylene oxide, PEO) with the aim to distinguish between free anions, ion pairs, and more complex ion aggregates [Gejji et al. (1993); Hu et al. (2005); Huang et al. (1994); Caruso et al. (2002)]. The interpretation of the frequency shifts in the Raman spectrum, however, has not been straightforward since both geometry and proximity influence the direction of the shift for these vibrations (red shift or blue shift). Nevertheless, the frequency of a stretching mode correlates directly with the strength of the molecular bond; hence a red shift indicates an elongation of the chemical bond and *vice versa* for a blue shift. Based on this, our results indicate that, on average, the S-O bond elongates while the C-F bond shortens as the protic ionic liquid [C_2_HIm][TFSI] is added to [C_2_HIm][TfO], Figure 8. Moreover, although distinct, the recorded shifts are limited to a few wavenumbers (3,031–3,034 cm^−1^ and 759–761 cm^−1^) within the whole compositional range, suggesting that the TfO^−^ remains in the state of a free anion. Indeed, based on the available literature, if the TfO^−^ anion had changed to be part of a contact ion pair or a more complex aggregate, distinct new modes at ∼1,042 cm^−1^ and ∼1,052 cm^−1^ will have been observed (Huang et al. (1994); Caruso et al. (2002); Hu et al. (2005)), which is clearly not the case in this study. Interestingly, the vibrational mode at 742 cm^−1^, a strong signature of the TFSI^−^ anion sensitive to intermolecular interactions, also red shifts as the protic ionic liquid [C_2_HIm][TfO] is mixed to [C_2_HIm][TFSI] (see Supplementary Figure S8). Thus, both anions are affected by composition in the way they interact with the cation, and the mixtures cannot be seen as simple weighted averages of the two pure protic ionic liquids. In light of these results, the overall blue shift of the N-H stretching mode observed by infrared spectroscopy as more TFSI^−^ is added, can be explained by the concomitant increase of [C_2_HIm][TFSI] species and the weakening of interactions within the already present [C_2_HIm][TfO] species. These trends, along with the phase behavior shown by DSC, indicate that upon mixing these two protic ionic liquids a more disordered and less strongly coordinated ionic system is created.

### 3.3 Ionic Conductivity

**FIGURE 9:**
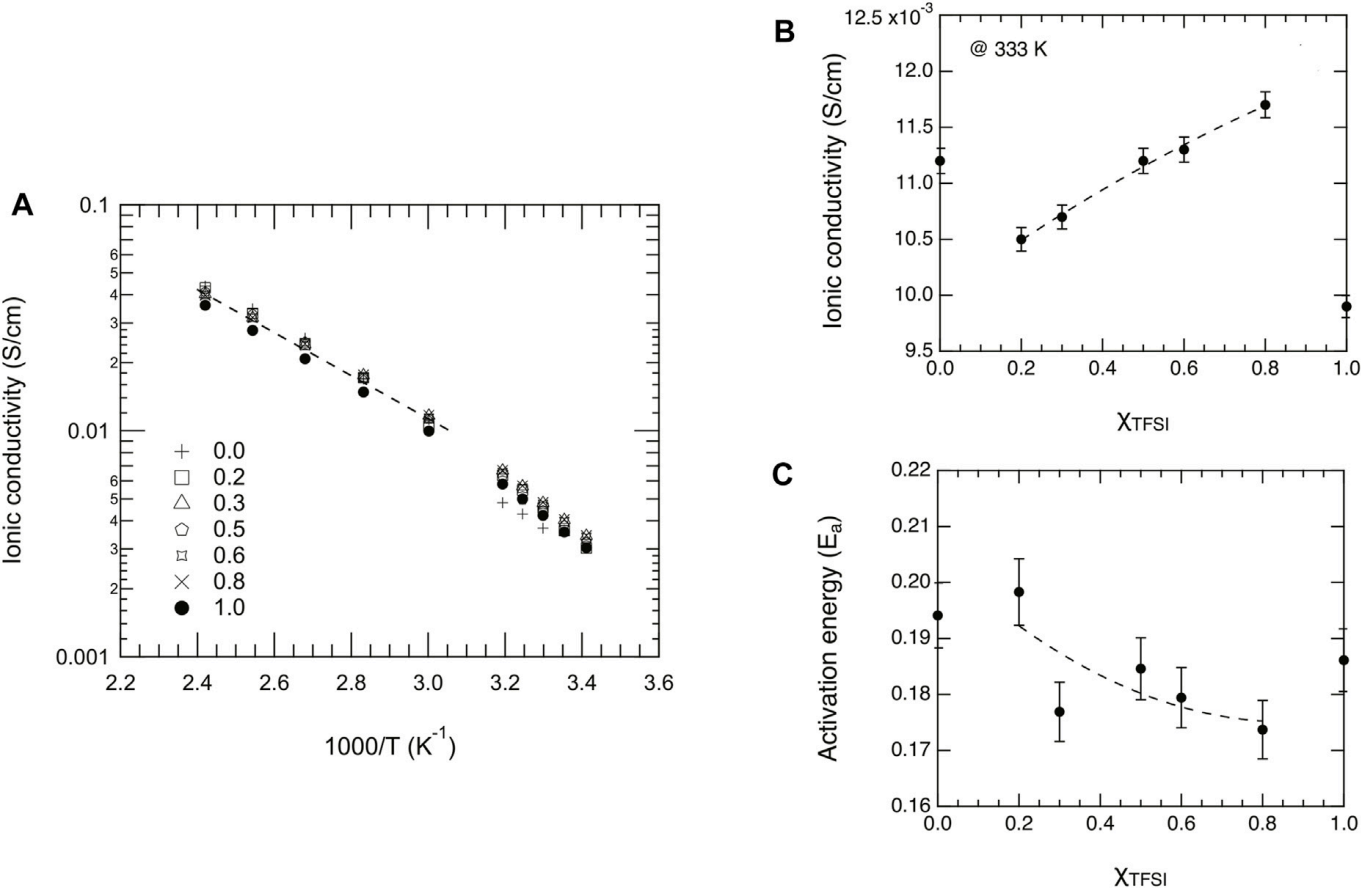
**(A)** Arrhenius plot of the dc ionic conductivity as a function of reciprocal temperature and for different mole fractions, *χ*, of [C_2_HIm][TFSI]. **(B)** Ionic conductivity extrapolated for the different liquids at the temperature of 333 K. **(C)** Activation energy, E_*a*_, as a function of composition (*χ*_*TFSI*_) estimated from fitting a limited number of data points in the high temperature limit.

The ionic conductivity of liquid materials can be measured by electrochemical impedance spectroscopy using a simple dip probe or a more advanced broadband dielectric spectrometer. The latter is also known as BDS and allows for measuring a variety of dielectric properties of materials covering a wide range of frequencies, typically in the range of 10^−1^–10^−7^ Hz, and large temperature windows (down to the temperature of liquid nitrogen) (Woodward (2021)). The ionic conductivity of the liquids investigated in this work was measured by BDS, covering wide frequency and temperature windows. The frequency dependence of the real part of conductivity, *σ*′(*ν*), is presented in Supplementary Figure S9 for temperatures between 20 and 140°C. All the plots reveal the frequency dependence typically observed in ionic liquids and other ion-conducting materials (Sippel et al. (2015); Shojaatalhosseini et al. (2017)). For all mixtures, a plateau is observed at frequencies approximately between 10^3^ and 10^6^ Hz, from which the direct current ionic conductivity (*σ*_*dc*_) can be extrapolated. At frequencies lower than 10^3^ Hz, *σ*′ decreases due to the known effect of electrode polarization (Sangoro et al. (2011); Sangoro et al. (2014); Sippel et al. (2015)). This is typically associated to an upturn of the real part of permittivity, *ϵ*’(*ν*) (see also Supplementary Figure S10), that in ionic liquids is anomalously large (Sippel et al. (2015)). The plateau values, i.e. the values of *σ*_*dc*_, increase with temperature as expected and are all shown in the Arrhenius plot of Figure 9. These values fall within the range 1⋅10^–3^–4⋅10^–1^ S/cm for temperatures between 20 and 140°C. An exception is the case of the pure ionic liquid [C_2_HIm][TfO] (*χ* = 0), which shows a discrete jump in conductivity below 30°C (see also Figure 9A) which is simply the effect of a first-order phase transition from the solid to the liquid state, congruent with the DSC trace shown in Figure 3 and the related phase diagram discussed previously. As proposed by Luo et al. (Luo et al. (2013)) while studying the ionic liquid imidazolium methanesulfonate, the increase in ionic conductivity with temperature may be explained by a more disordered system (with possible contributions from rotational freedom) that creates vacancies facilitating ionic motion.

For a deeper interpretation of these experimental data, a selected number of data points have been fitted with the Arrhenius equation $\sigma =\sigma_{0}\cdot exp\left(\frac{-Ea}{k_{B}T}\right)$ (Moynihan et al. (1982)) in order to estimate the activation energy, E_*a*_, of ionic conduction.^2^ The last five points in the high-temperature range have been selected, to be safely away from temperatures of possible solid-to-liquid transitions. The use of the Arrhenius equation is motivated by the selection of a limited number of points, while the use of the VFT (Vogel–Fulcher–Tammann) equation would have been more appropriate if experimental data had been collected down to much lower temperatures, approaching the glass transition temperatures T_*g*_. The dynamical properties at extremely low temperatures, however, was not in the scope of this study. The values of E_*a*_ that we find do not vary significantly with composition and fall within the range 0.17–0.21 eV (see also Table 1). In magnitude, these values are similar to those reported in the work of Bulut *et al.* (0.19–0.26 eV) (Bulut et al. (2011)), who studied the ionic conductivity of nine pure ionic liquids based on imidazolium and pyridinium cations (the anion being always TFSI^−^). In addition, the dc ionic conductivity as a function of composition has been investigated for the arbitrarily selected temperature of 60°C (Table 1). These values do not show significant variations either, which is reasonable given that the two protic ionic liquids used for mixing have conductivity values in their pure states very close to each other, e.g. 11.2 mS/cm for [C_2_HIm][TfO] and 9.9 mS/cm for [C_2_HIm][TFSI] at 60°C. Nevertheless, it is somehow interesting to observe that, within the small range of values found, both the ionic conductivity and the activation energy change monotonically with composition (if the data points of the pure ionic liquids are excluded), the former slightly increasing (Figure 9B) and the latter slightly decreasing (Figure 9C) instead. Although we judge that these trends (higher conductivity and lower E_*a*_) should be interpreted carefully, they may reasonably reflect the depression of both the melting and glass transition temperatures observed with DSC upon increasing *χ*_*TFSI*_.

## 4 Conclusion

A series of mixtures based on the protic ionic liquids 1-ethylimidazolium triflate and 1-ethylimidazolium bis(trifluoromethylsulfonyl)imide have been investigated, the cation being in common and the anions differing in size, symmetry, and pK_*a*_. Complementary experimental methods such as vibrational spectroscopy, calorimetry, and dielectric spectroscopy have been employed with the aim of correlating the dynamics and local structure. Our results reveal that upon increasing the content of TFSI^−^ in the mixture, the phase behavior changes; in particular, the melting temperature is first decreased (*χ* ≤ 0.3) and then suppressed (0.3 <χ≤ 0.8). Vibrational spectroscopy (combined Raman and infrared spectroscopies) indicate that as *χ*_*TFSI*_ increases, the average cation⋅⋅⋅anion interactions become weaker and both anions are affected by the presence of each other. The ionic conductivity is similar in the two pure ionic liquids and absolute values do not change much upon mixing. Nevertheless, within the range of the measured values, a trend with an increasing conductivity with *χ*_*TFSI*_ is noted, which may be a direct effect of the suppressed melting temperature. This seems to be accompanied by decreasing E_*a*_ values. To conclude, the most important implication of these results, obtained by three complementary techniques, is that a compositional range has been identified (0.3 <χ≤ 0.8) that provides a wide temperature window of the liquid state (in fact, no phase transitions at all) without compromising the ionic conductivity, which in turn can be rationalized by an established greater disorder at the molecular level. Hence, such mixtures can be used in electrochemical devices such as batteries, supercapacitors, or fuel cells, operating in a wide range of temperatures extending well above and below room temperature. An extension of the operational temperature window is essential for companies and is searched to ensure safety and use in remote areas with extreme climate conditions.

## Data Availability

The raw data supporting the conclusions of this article will be made available by the authors, without undue reservation.

## Footnotes

1 To calculate molalities, the following molecular weights have been used; 246.21 g/mol for C_2_HImTfO and 377.28 g/mol for C_2_HImTFSI.

2 In this Arrhenius equation, *σ* is the ionic conductivity (in S/cm), *σ*_0_ is the ionic conductivity extrapolated for infinite temperatures, E_*a*_ is the activation energy, k_*B*_ is the Boltzmann’s constant and T is the temperature (in K).

## Abbreviations

BDS, broadband dielectric spectroscopy; BF_4_, tetrafluoroborate; BMIM, butyl-methylimidazolium; Cl, chloride; C_2_HIm, 1-ethylimidazolium; CF_3_SO_3_, triflate; CH_3_SO_3_, methylsulfonate; dema, diethylmethylammonium; DES, deep eutectic solvent; DSC, differential scanning calorimetry; Et_3_NH, triethylammonium; FSI, bis(fluorosulfonyl)imide; HSO_4_, hydrogen sulfate; NMR, nuclear magnetic resonance; NTf_2_, bis(trifluoromethanesulfonyl)imide; PEM, proton exchange membrane; PEO, polyethylene oxide; PYR, pyrrolidinium; TfO, triflate; TFSI, bis(trifluoromethanesulfonyl)imide; VFT, Vogel–Fulcher–Tammann.
