# Supplementary material for: Binary Mixtures of Imidazolium-Based Protic Ionic Liquids. Extended Temperature Range of the Liquid State Keeping High Ionic Conductivities
Source: Front Chem. 2022 Jul 1;10:915683. doi: 10.3389/fchem.2022.915683 (PMC9283952; doi:10.3389/fchem.2022.915683)
Supplement: Supplementary file 1 [file DataSheet1.PDF]

Supplementary Material

1 SUPPLEMENTARY DATA

This file provides a set of supplementary information in the form of tables or figures, that complement the information already given in the main text of the article.

2 SUPPLEMENTARY TABLES AND FIGURES

Table S1. Phase behaviour, Ionic conductivity (at 333 and 393 K; i.e at 60 and 120 °C) and activation energy for ionic conduction (E_a) as a function of composition (χ_{TFSI})

χ_{TFSI}	T_c (°C)	T_m (°C)	H_m (J/g)	σ @ 60 °C (mS/cm)	σ @ 120 °C (mS/cm)	E_a (eV)
0.0	-16	28.8	66	11.2	34.6	0.1941
0.1	-41; -25	19.9	55	5.4	21.0	0.2116
0.2	-43	13.7	44	10.5	33.0	0.1983
0.3	a	3.8	3	10.7	27.4	0.1769
0.5	a	-	-	11.2	32.7	0.1846
0.6	a	-	-	11.3	31.6	0.1794
0.8	a	-	-	11.7	32.9	0.1737
1.0	-30	-0.4	24	9.9	27.8	0.1861

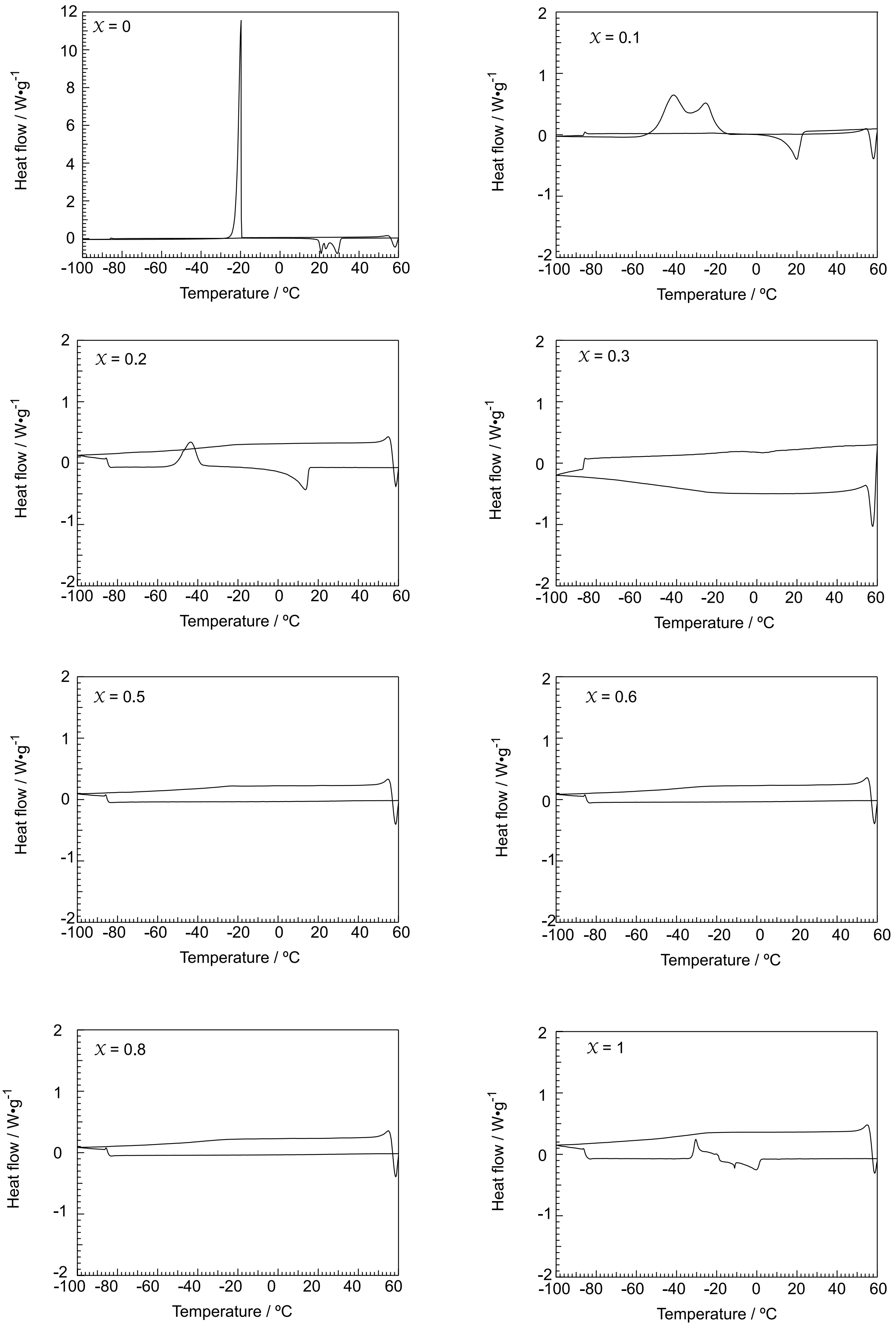

Figure S1. DSC thermograms of the binary ionic liquids mixtures at different mole fractions of [C_2HIm][TFSI], recorded during the second cooling and heating scans. Endothermic peaks down.

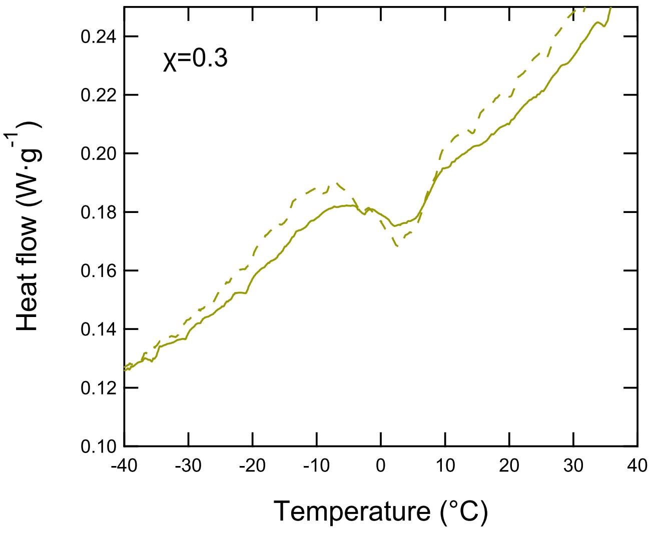

Figure S2. DSC thermograms of the binary ionic liquids mixtures with χ=0.3, recorded during the second cooling and heating scan. Endothermic peaks down.

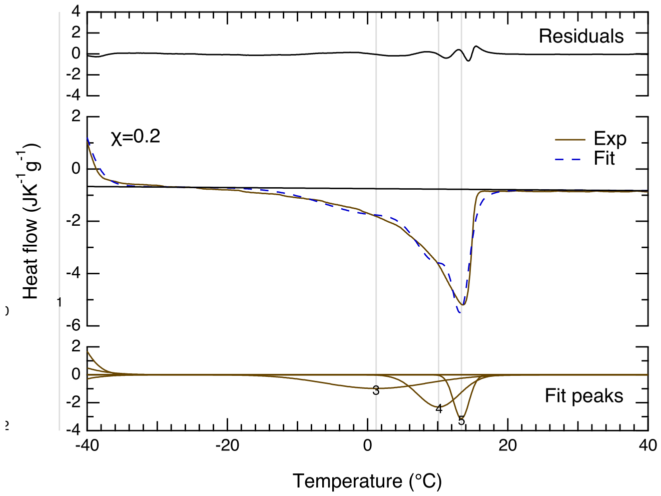

Figure S3. DSC thermogram of the binary ionic liquids mixture with χ=0.2, recorded during the second heating scans (endothermic peaks down), shown together with a fitting model.

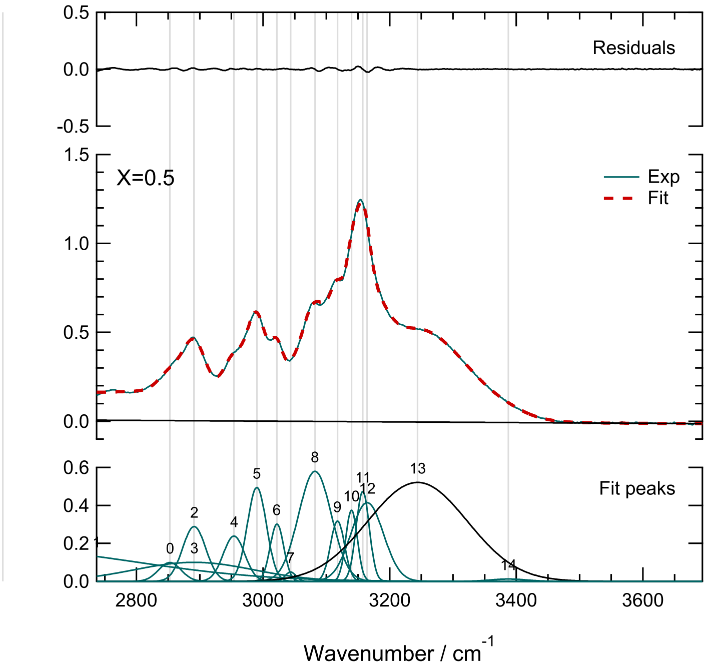

Figure S4. Example of the peak fit procedure applied to infrared spectra, here for the case of the mixture with $\chi = 0.5$.

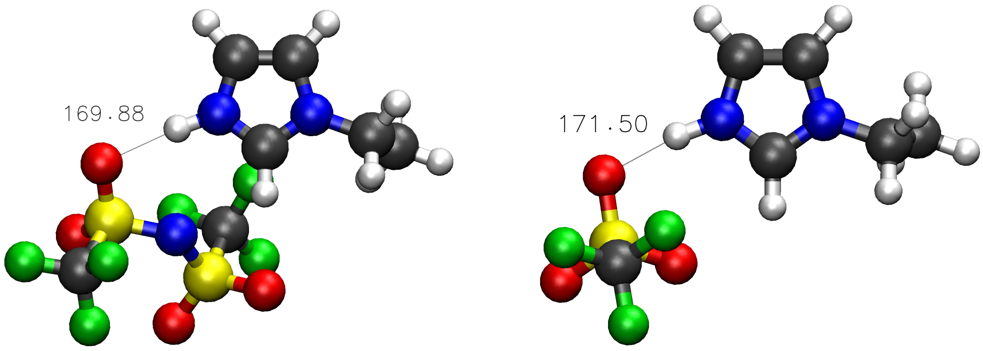

Figure S5. Lowest energy configurations computed for the two protic ionic liquids and single ion pairs; [C_2HIm][TFSI] to the left and [C_2HIm][TfO] to the right. These DFT calculations have been performed by Eduardo Maurina Morais, a PhD student in the research group of Anna Martinelli.

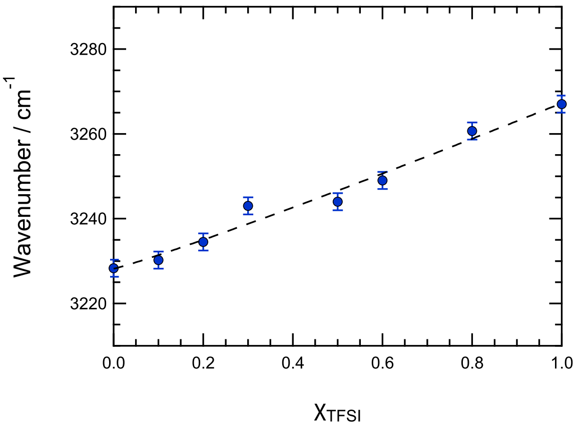

Figure S6. Composition dependence of the N-H stretching mode recorded by infrared spectroscopy at ambient temperature and pressure conditions.

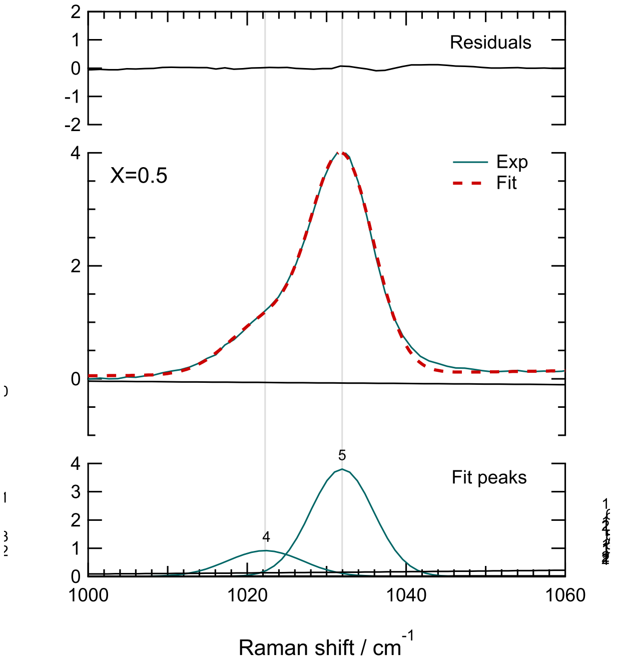

Figure S7. Example of the peak fit procedure applied to Raman spectra, here for the case of the mixture with $\chi = 0.5$.

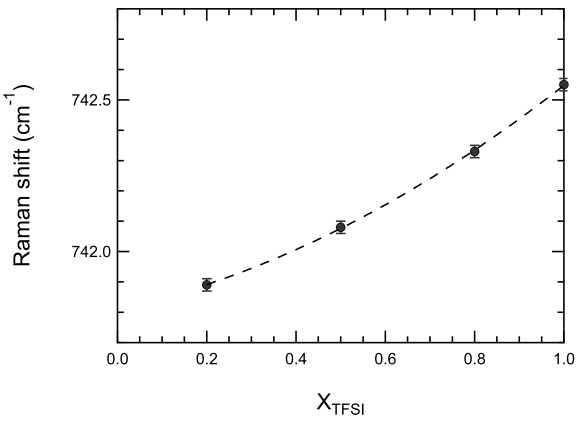

Figure S8. Raman shift of the vibrational mode characteristic of TFSI at 742 cm^{-1} as a function of composition.

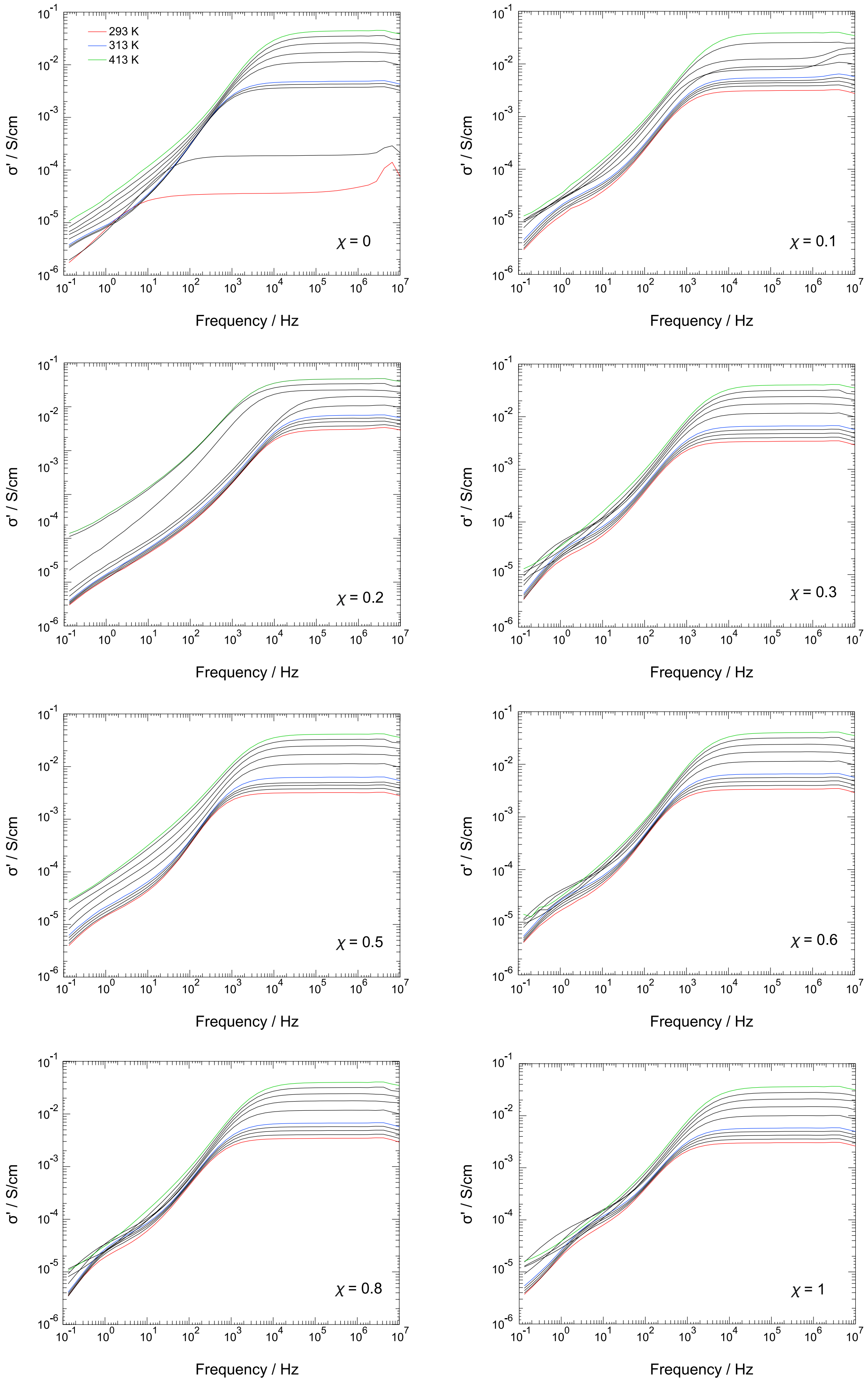

Figure S9. Frequency dependence of the real part of ionic conductivity of the binary ionic liquids mixtures measured at different temperatures.

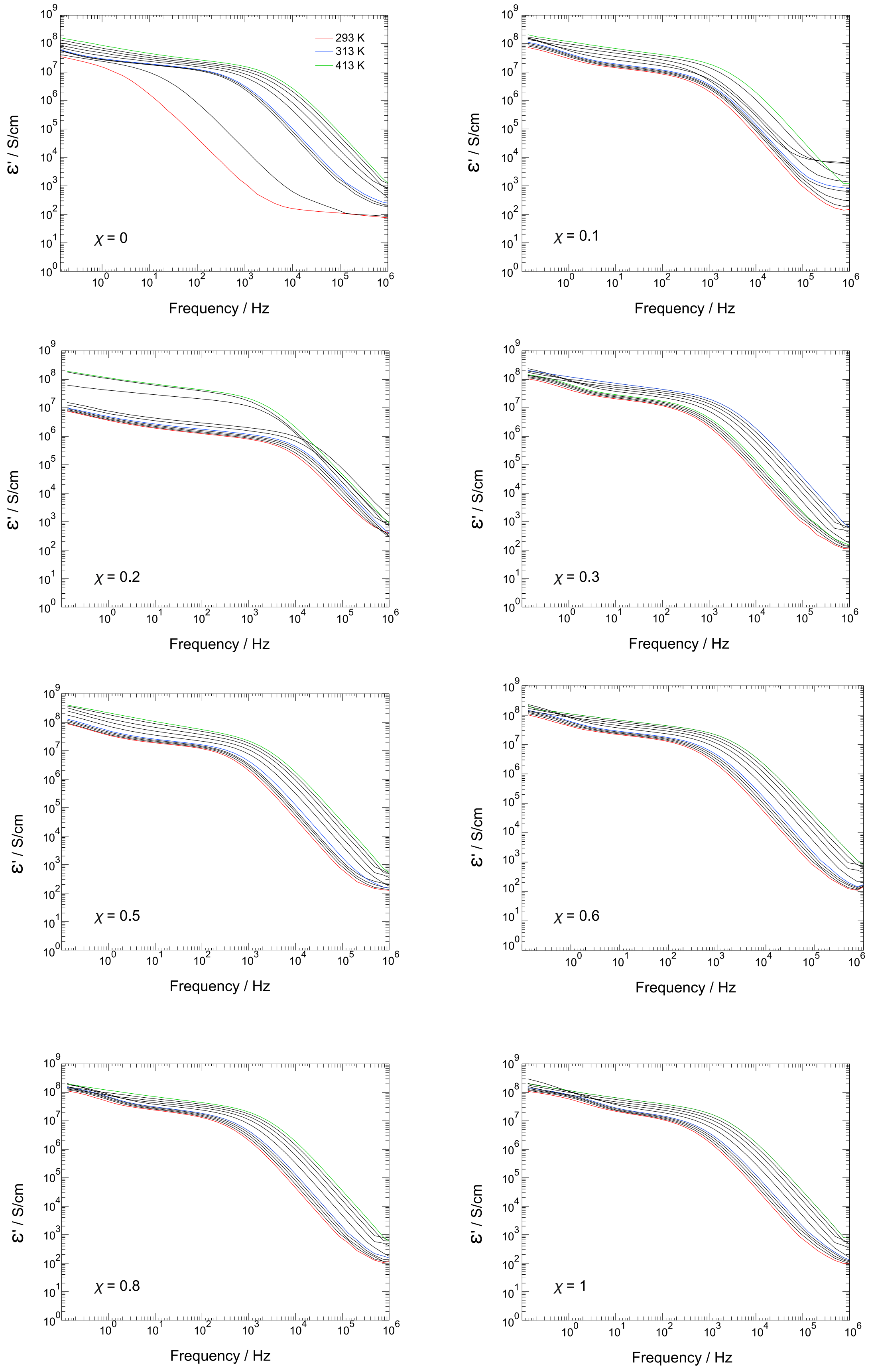

Figure S10. Frequency dependence of the real part of permittivity of the binary ionic liquids mixtures measured at different temperatures.
